# Editorial: Applications of geospatial information technologies and spatial statistics in health services research

**DOI:** 10.3389/fpubh.2023.1349985

**Published:** 2024-01-04

**Authors:** Chao Song, Xiuli Wang, Erjia Ge, Xun Shi, Jay Pan

**Affiliations:** ^1^HEOA Group, West China School of Public Health and West China Fourth Hospital, Sichuan University, Chengdu, China; ^2^West China-PUMC C.C. Chen Institute of Health, Sichuan University, Chengdu, China; ^3^Institute for Healthy Cities and West China Research Centre for Rural Health Development, Sichuan University, Chengdu, China; ^4^Dalla Lana School of Public Health, University of Toronto, Toronto, ON, Canada; ^5^Department of Geography, Dartmouth College, Hanover, NH, United States; ^6^China Center for South Asian Studies, Sichuan University, Chengdu, China

**Keywords:** health econometrics, spatial econometrics, spatial health statistics, health and medical geography, Geographic Information Science

The integration of geospatial information technologies and spatial statistical methodologies into health services research constitutes a paradigm shift from conventional non-spatial analyses that have historically overlooked the pivotal laws of geography (1). Over the last decade, a remarkable expansion has occurred within the realm of health economics and public health, specifically in the adoption of advanced geospatial technologies and sophisticated spatial health statistical techniques (2). This editorial seeks to highlight and critically appraise some of the cutting-edge applications of these technologies and methods, elucidating their significance in addressing contemporary public health challenges, particularly within health services research (3).

Within the scope of health services research, two critical dimensions—spatial equity and spatial accessibility—have garnered significant attention (4). This Research Topic features four pivotal articles that delve into these dimensions. In the study “*Measuring the inequalities in healthcare resource in facility and workforce: a longitudinal study in China*,” Dong et al. investigate the nuanced aspects of spatial inequality in healthcare resource distribution, addressing both the propensity toward equalization and the tendency for geographic agglomeration. In light of their work, we advocate for a shift in focus toward fine-grained, small-area analyses that are simultaneously precise and holistic, advocating for multidimensional assessments that consider driving factors, spatiotemporal dynamics, and the integrative evaluation of various indicators (5, 6).

On the matter of spatial healthcare accessibility, Hua et al., in “*Are the epidemic prevention facilities effective? How cities should choose epidemic prevention facilities: taking Wuhan as an example*,” utilize travel time as a core measure of accessibility for essential and emergency epidemic prevention facilities. Notably, the Two-Step Floating Catchment Area (2SFCA) method, originally conceptualized by Wang, stands as the benchmark for evaluating spatial accessibility within the healthcare sector (7). Building upon this, Wang et al., in “*Disparities in spatial accessibility of primary care in Louisiana: from physical to virtual accessibility*,” introduce the novel Two-Step Virtual Catchment Area (2SVCA) method. This innovative approach assesses the spatial accessibility of primary care services through telehealth, thus expanding the concept to embrace virtual accessibility. Furthermore, Molenaar et al., in “*Travel scenario workshops for geographical accessibility modeling of health services: a transdisciplinary evaluation study*,” establish an essential premise that enhancing and standardizing knowledge elicitation processes for the creation of realistic travel scenarios, encompassing transportation modes and velocities, is crucial for accurately calculating geographical access to health services.

The trifecta of geo-environmental big data, Geographic Information Science (GIS), and spatial statistics stands at the forefront of contemporary health research, paving the way for groundbreaking insights and advancements. The three remaining articles in this Research Topic encapsulate the innovative application of these tools in elucidating complex health-related issues.

In “*Comparison of the association between different ozone indicators and daily respiratory hospitalization in Guangzhou, China*,” Lin et al. utilize a spatial interpolation method to transform point-specific environmental data into a continuous spatial field. This process facilitates the estimation of health impacts by correlating environmental data with the residential locations of patients. It is noteworthy that the emergence of advanced geo-environmental data products derived from satellite remote sensing technologies now obviates the need for individual researchers to perform such interpolations, offering readily applicable data for health research (8, 9).

Lu and Ren's review article, “*Diseases spectrum in the field of spatiotemporal patterns mining of infectious diseases epidemics: a bibliometric and content analysis*,” underscores the critical role and extensive utility of GIS technologies and spatial statistics within the domain of infectious disease research. It identifies human mobility and scale effects as pivotal areas for future exploration. Here, we further accentuate the imperative for methodological innovation in the integrated analysis of space-time dynamics, advocating for advancement beyond the traditional compartmentalization of these dimensions (10).

Tang et al. in “*A spatiotemporal analysis of the association between carbon productivity, socioeconomics, medical resources, and cardiovascular diseases in southeast rural China*,” present a sophisticated case study employing both the Multiscale Geographically Weighted Regression (MGWR) and the Geographically and Temporally Weighted Regression (GTWR) models, which embody the second law of geography, to account for the spatial and temporal heterogeneity inherent in health outcomes and their determinants. Frequency statistics and Bayesian statistics are the two main schools of statistics. The frequentist GTWR model is juxtaposed with the Bayesian Spatiotemporally Varying Coefficients (STVC) model (11, 12), which represents a unified full-map framework for detecting heterogeneous relationships over space and time, and has also been successfully implemented in health and medical geography studies (13).

This Research Topic offers a view of the recent paradigmatic achievements in the intersecting realms of geospatial technologies and health research. Although the compilation here represents but a fraction of the burgeoning field, it is envisaged that the research presented will act as seminal references, stirring academic curiosity and innovation. The advent of big geospatial data has revolutionized the study of human health, imposing new challenges upon the methods employed in geospatial health and medical research. The emerging fields of Geospatial Artificial Intelligence (GeoAI), geospatial causal inference (14), coupling of individual and regional health data (15), population movement trajectories, scale effects, and multi-scale spatiotemporal heterogeneity modeling (10), are just a few areas where further methodological evolution is both anticipated and essential.

In anticipation of the future, we invite scholars—including public health experts, GIS practitioners, and spatial statisticians—to engage with these challenges, fostering the development of novel methodologies designed to address the sophisticated and intricate scientific queries that lie at the intersection of public health, geography, and big data analytics. It is through such interdisciplinary collaboration and innovation that the field will continue to expand and contribute to the betterment of global health outcomes.

## Author contributions

CS: Conceptualization, Funding acquisition, Project administration, Writing—original draft. XW: Funding acquisition, Writing—review & editing. EG: Writing—review & editing. XS: Writing—review & editing. JP: Conceptualization, Funding acquisition, Writing—review & editing.
